# Fifty-Eighth Annual Meeting of the New York State Medical Society

**Published:** 1865-02

**Authors:** 


### EDITORIAL DEPARTMENT.

### FIFTY-EIGHTH ANNUAL MEETING OF THE NEW YORK STATE MEDICAL SOCIETY.

Dr. C. C. WYCKOFF, delegate from Erie County Medical Society has kindly furnished us with the report of the transactions of the State Medical Society, as published in the Albany *Atlas and Argus*, from which we extract the following items of business, leaving the names of the medical papers which were presented, for consideration after their publication; when their merits may be more fully determined:

TUESDAY Feb. 7. 1865.

The New York State Medical Society met pursuant to statute, in the City Hall, this morning at 11 o'clock. The meeting was called to order by the President of the Society, Dr. Fred. Hyde.

Ths President then delivered the opening address.

The Secretary announced an invitation from Governor Fenton to visit the Executive Mansion on Wednesday evening at 9 o'clock. Accepted.

Nominating Committee presented the following report:

The Nominating Committee would report the following persons:

For President—HENRY W. DEAN, Rochester.

For Vice President—JOSEPH C. HUTCHISON, Brooklyn.

For Secretary—SYLVESTER D. WILLARD.

For Treasurer—JOHN V. L. QUACKENBUSH.

The Secretary announced that he had received a report from Dr. Charles A. Lee, Chairman of the Committee on "Medical Education." The report was read, and on motion, accepted.

Dr. Brinsmade called up the following report, offered at the last annual meeting of the Society, and which was made a special order for twelve o'clock to-day:

"The undersigned, appointed a Committee to report upon a resolution passed by the Medical Society of the County of New York, relative to the propriety of Medical Practitioners advertising their specialty in medical or other journals, and referred to this Society for decision, beg leave to offer the following resolutions:

*Resolved*, That in the opinion of this Society it is impossible to define the limits of medical specialties, either in medical or other journals.

*Resolved*, That advertisements indicating location and residence, are the utmost limits of self-announcements, consistent with professional dignity; and that all reference to special branches of medical practice, as extra inducement to patronage, should be deemed violations of the Code of Medical Ethics.

*Resolved*, That hereafter any medical practitioner so offending shall be deemed disqualified as delegate to or for membership of this Society, and if already a delegate to, or a member thereof, shall be deemed a fit subject for discipline.

*Resolved*, That this Society recommends all Medical Societies in the State of New York to adopt the foregoing resolutions, with a view to establish the true dignity of our profession.

*Resolved*, That the foregoing resolutions be transmitted to the American Medical Association, at its next meeting, as an expression of the opinion of the Medical Society of the State of New York, and that for this purpose a committee of presentation be appointed.

Thomas C. Brinsmade, Howard Townsend, Guido Furman, Committee.

Dr. Staats moved the adoption of the report. Agreed to.

The Secretary offered the following, in behalf of Dr. Ira Harris, of New York:

*Whereas*, The prevalence of infectious diseases in the city of New York is a source of danger to the health of cities and towns throughout the State; and

*Whereas*, Our professional brethren in New York have put forth renewed and earnest efforts to promote a proper investigation of the sanitary question and to procure the establishment of a suitable system of sanitary government.

*Resolved*, That the New York State Medical Society hereby expresses its cordial interest in the efforts that are being made by leading physicians and citizens of New York to procure the enactment of an adequate system of sanitary government for that city.

*Resolved*, That it is the earnest wish and recommendation of this Society that the bill now pending before the Legislature, entitled "An Act to establish a Metropolitan Sanitary District and Board of Health, to preserve the public health to said district, and to prevent the spread of disease therefrom," be speedily enacted with a law.

*Resolved*. That a copy of these resolutions be presented to the presiding officers of the Senate and Assembly, respectively, with the request that the same be laid before those honorable bodies.

Dr. Kennedy called up the following resolutions, offered yesterday:

*Whereas*, The prevalence of infectious diseases in the city of New York is a source of danger to the health of cities and towns throughout the State; and

*Whereas*, Our professional brethren in New York have put forth renewed and earnest efforts to promote a proper investigation of the sanitary question, and to procure the establishment of a suitable system of sanitary government.

*Resolved*, That the New York State Medical Society hereby expresses its cordial interest in the efforts that are being made by leading physicians and citizens of New York to procure the enactment of an adequate system of sanitary government for that city.

*Resolved*, That it is the earnest wish and recommendation of this Society that the bill now pending before the Legislature, entitled "An Act to establish a Metropolitan Sanitary District and Board of Health, to preserve the public health in said district, and to prevent the spread of disease therefrom," be speedily enacted into a law.

*Resolved*, That a copy of these resolutions be presented to the presiding officers of the Senate and Assembly, respectively, with the request that the same be laid before those honorable bodies.

After discussion, the resolutions were unanimously adopted.

The Secretary, in behalf of Dr. Orton, presented the following, which were adopted:

*Resolved*, That a standing committee on statistics be annually appointed by this Society, whose duties shall be to solicit, collect and preserve documents relating to the medical profession, and the practice of medicine and surgery in the State of New York.

*Resolved*, That the said Committee prepare a circular, which shall be submitted to and issued by the Committee of Publication of this Society, inviting correspondence and the transmission of facts and observations in the various departments of professional inquiry.

*Resolved*, That the Committee consist of eight members, and shall be selected one from each Senatorial District.

Dr. Steward offered the following, which was adopted:

*Resolved*, That practicing physicians who habitually fail for four consecutive years to join or neglect to attend the meetings of the County Medical Society in which such persons may reside, or some other regular and legal medical organization, in said County, are not eligible to a seat in this body, either by invitation or otherwise.

Dr. Brinsmade offered the following:

*Resolved*, That the thanks of the Society be extended to Dr. Hyde for his very able and interesting address. Adopted.

The Secretary read the following from Dr. T. C. Brinsmade, of Troy:

"I will give $100 for the best essay on medical and vital statistics. The essay must be accompanied by a plan for making and tabulating hospital reports, records of private practice in medicine, surgery and obstetrics, together with a draft of a law for the registration of births, marriages and deaths. The prize to be awarded by the Committee of the New York State Medical Society on Prize Essays. The essay to be placed in the hands of the Committee, with the plans and drafts, on or before the 15th of December next."

Dr. Wyckoff offered the following, which was adopted:

*Resolved*, That a committee of one or more be appointed to inquire into the status of the law in regard to the reception of members into County Societies, and of the duty of the President in regard to notifying irregular practitioners to join the same.

After adopting a vote of thanks to the retiring officers, the Society adjourned *sine die*.

